# Virologic Monitoring of Poliovirus Type 2 after Oral Poliovirus Vaccine Type 2 Withdrawal in April 2016 — Worldwide, 2016–2017

**DOI:** 10.15585/mmwr.mm6620a4

**Published:** 2017-05-26

**Authors:** Ousmane M. Diop, Humayun Asghar, Evgeniy Gavrilin, Nicksy Gumede Moeletsi, Gloria Rey Benito, Fem Paladin, Sirima Pattamadilok, Yan Zhang, Ajay Goel, Arshad Quddus

**Affiliations:** ^1^World Health Organization (WHO), Geneva, Switzerland; ^2^Eastern Mediterranean Regional Office, WHO; ^3^European Regional Office, WHO; ^4^African Regional Office, WHO; ^5^Americas Regional Office, WHO; ^6^South East Asian Regional Office, WHO; ^7^Western Pacific Regional Office, WHO.

The Global Polio Eradication Initiative (GPEI) has made substantial progress since its launch in 1988; only 37 wild poliovirus type 1 (WPV1) cases were detected in 2016, the lowest annual count ever. Wild poliovirus type 3 has not been detected since November 2012, and wild poliovirus type 2 was officially declared eradicated in September 2015. This success is attributable to the wide use of live oral poliovirus vaccines (OPVs). Since 2001, numerous outbreaks were caused by the emergence of genetically divergent vaccine-derived polioviruses (VDPVs) whose genetic drift from the parental OPV strains indicates prolonged replication or circulation (*1*). In 2015, circulating VDPV type 2 (cVDPV2) outbreaks were detected in five countries worldwide (Nigeria, Pakistan, Guinea, Burma, and South Sudan), and VDPV2 single events were reported in 22 countries. These events prompted the GPEI to withdraw the type 2 component (Sabin2) of trivalent OPV (tOPV) in a globally coordinated, synchronized manner in April 2016 (*2*,*3*), at which time all OPV-using countries switched to using bivalent OPV (bOPV), containing Sabin types 1 and 3. This report details for the first time the virologic tracking of elimination of a live vaccine that has been withdrawn from routine and mass immunization systems worldwide (*3*). To secure elimination, further monitoring is warranted to detect any use of tOPV or monovalent OPV type 2 (mOPV2).

## The Global Polio Laboratory Network

The Global Polio Laboratory Network (GPLN) comprises 146 World Health Organization (WHO)–accredited poliovirus laboratories in 92 countries located in the six WHO regions (*4*). GPLN member laboratories follow standardized protocols to isolate poliovirus using sensitive and specific cell lines, conduct intratypic differentiation to identify WPVs, Sabin (vaccine) polioviruses, or screen for VDPVs, and conduct genomic sequencing. Sequencing results help monitor pathways of poliovirus transmission by comparing the nucleotide sequences of the capsid protein VP1-coding regions of poliovirus isolates. The GPLN processes approximately 200,000 specimens from cases of acute flaccid paralysis (AFP) each year and provides timely results to direct GPEI actions. The accuracy and quality of testing at GPLN member laboratories is monitored through an annual accreditation program that includes on-site reviews of work practices, performance, and proficiency testing (*5*).

## Surveillance Systems

GPLN laboratories provide support to different polio surveillance systems, including AFP surveillance, environmental surveillance (testing of sewage samples), and enterovirus surveillance (testing of patients with specific clinical illness caused by enteroviruses). These surveillance systems ensure sensitive and timely detection of circulating polioviruses worldwide. Whereas AFP surveillance has been the standard surveillance system for poliovirus since the beginning of the GPEI, recently, existing environmental surveillance for poliovirus has been expanded (*6*) in countries with endemic poliovirus transmission and in countries designated as countries at high risk for WPV importation and circulation and/or VDPV emergence. During the last 5 years, 11 laboratories dedicated to environmental surveillance were established in Bangladesh, Cameroon, Côte d’Ivoire, Senegal, South Africa, Indonesia, Jordan, Kenya, Madagascar, Niger, and the Philippines; equipment and supplies were procured by WHO and field and laboratory personnel were trained by GPLN (*7*). This infrastructure, combined with the existing environmental surveillance system and AFP surveillance, has been used to monitor Sabin type 2 virus circulation after worldwide OPV2 withdrawal in April 2016.

## Detection of Type 2 Polioviruses

Before OPV2 withdrawal, mass immunization campaigns using tOPV were conducted in OPV-using countries, to ensure that sufficiently high levels of immunity against poliovirus type 2 (PV2) were achieved in all countries. From January to April 2016 (before the global switch from tOPV to bOPV), 46 countries were reporting PV2 detected by GPLN laboratories from specimens from persons with AFP or their contacts and sewage samples (Table). From May to August 2016 (during the early switch period), the number of countries reporting PV2 declined to 22; from September to December 2016, eight countries reported isolation of PV2, and from January to March 2017, seven countries (Afghanistan, Cameroon, Chad, Mozambique, Niger, Nigeria, and Pakistan) reported PV2 detection.

**TABLE:** Countries that have reported isolating poliovirus type 2 (PV2) from persons with acute flaccid paralysis or their contacts and from sewage samples, January 2016–March 2017

Countries	Human specimens				Sewage samples			
	2016			2017	2016			2017
	Jan–Apr (pre-switch	May–Aug (early post-switch)	Sep–Dec (post-switch)	Jan–Mar	Jan–Apr (pre-switch)	May–Aug (early post-switch)	Sep–Dec (post-switch)	Jan–Mar
**mOPV used post-switch (six countries)**								
Cameroon	4	—	1	14	—	—	—	—
Chad	3	—	—	7	—	—	—	1
Mozambique	—	—	—	1	—	—	—	—
Niger	8	—	1	6	—	—	—	1
Nigeria	341	64	26	103	123	65	24	196
Pakistan	42	4	—	5	99	14	3	29
**No. of countries/No. of isolates**	5/398	2/68	3/28	6/136	2/222	2/79	2/27	4/227
**mOPV not used post-switch (44 countries)**								
Afghanistan	22	1	1	—	16	—	—	1
Algeria	1	—	—	—	—	—	—	—
Angola	1	—	—	—	—	—	—	—
Azerbaijan	1	—	—	—	—	—	—	—
Bahrain	1	—	—	—	—	—	—	—
Bangladesh	1	—	—	—	—	—	—	—
Benin	1	—	—	—	—	—	—	—
Bhutan	1	—	—	—	—	—	—	—
Bosnia and Herzegovina	1	3	—	—	—	—	—	—
Burkina Faso	15	—	—	—	—	—	—	—
Burma	2	—	—	—	—	—	—	—
Central African Republic	2	1	—	—	—	—	—	—
Republic of the Congo	1	1	—	—	—	—	—	—
Côte d’Ivoire	5	—	—	—	—	—	—	—
Democratic Republic of the Congo	54	—	—	—	—	—	—	—
Egypt	8	1	—	—	—	—	—	—
Ethiopia	15	2	—	—	—	—	—	—
Guinea	40	3	—	—	—	—	—	—
India	345	7	—	—	13	53	4	—
Indonesia	15	1	—	—	—	—	—	—
Iran	—	1	—	—	—	—	—	—
Iraq	20	—	2	—	—	—	—	—
Israel	—	—	—	—	1	—	—	—
Kazakhstan	—	—	—	—	2	—	—	—
Kenya	3	—	—	—	22	3	—	—
Madagascar	26	—	—	—	6	10	—	—
Moldova	—	—	—	—	1	3	—	—
Mali	11	—	—	—	—	—	—	—
Morocco	1	—	—	—	—	—	—	—
Nepal	2	—	—	—	—	—	—	—
Russia	7	4	2	—	3	5	1	—
Senegal	2	—	—	—	—	—	—	—
Sierra Leone	2	—	—	—	—	—	—	—
Somalia	7	2	—	—	—	—	—	—
South Sudan	10	5	—	—	—	—	—	—
Sudan	—	1	—	—	—	—	—	—
Syria	—	1	—	—	—	—	—	—
Thailand	1	—	—	—	—	—	—	—
Turkmenistan	2	—	—	—	—	—	—	—
Uganda	13	—	—	—	—	—	—	—
Ukraine	5	—	—	—	5	1	—	—
Tanzania	2	—	—	—	—	—	—	—
Yemen	5	2	—	—	—	—	—	—
Zimbabwe	1	—	—	—	—	—	—	—
**No. of countries/No. of isolates**	**38/652**	**16/36**	**3/5**	**0/0**	**9/69**	**6/75**	**2/5**	**1/1**
**Total all countries/All isolates**	**43/1,050**	**18/104**	**6/33**	**6/136**	**11/291**	**8/154**	**4/32**	**5/228**

**Abbreviation:** mOPV2 = monovalent oral poliovirus vaccine type 2.

Field investigations in response to detection of PV2 after the switch found breaches in OPV2 withdrawal with evidence of continued inadvertent use of tOPV in India (*8*), Pakistan, Afghanistan, Russia, Iraq, Nigeria, and Cameroon. Response to these breaches included development of guidelines for investigation and implementation of corrective actions to ensure the safe disposal of all tOPV vials. For example, in India, the National Polio Surveillance Program established a policy to replace any tOPV vial found in private clinics with two bOPV vials as an incentive for finding and reporting tOPV vials. All countries with PV2 detected in 2017 (except Afghanistan) conducted immunization campaigns using monovalent oral poliovirus vaccine type 2 (mOPV2) in response to cVDPV2 isolates detected in Pakistan and Nigeria. PV2 detected in Afghanistan was linked to the use of mOPV2 in a neighboring district of Pakistan.

**FIGURE 1:**
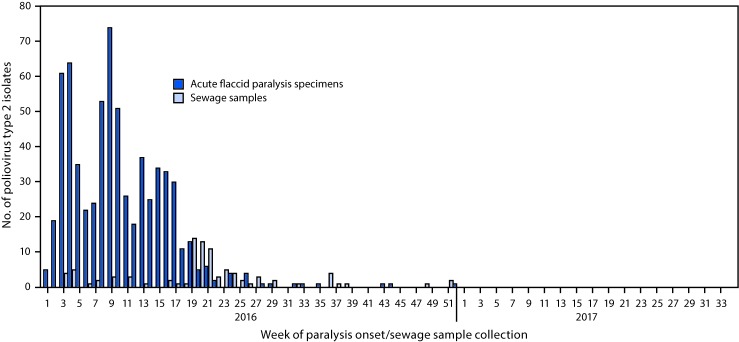
Number of poliovirus type 2 isolates from persons with acute flaccid paralysis or their contacts and from sewage samples in countries where mOPV2 was not used after the global synchronized switch from tOPV to bOPV — January 2016–March 2017 **Abbreviations:** bOPV = bivalent oral poliovirus vaccine; mOPV2 = monovalent oral poliovirus vaccine type 2; tOPV = trivalent oral poliovirus vaccine.

**FIGURE 2:**
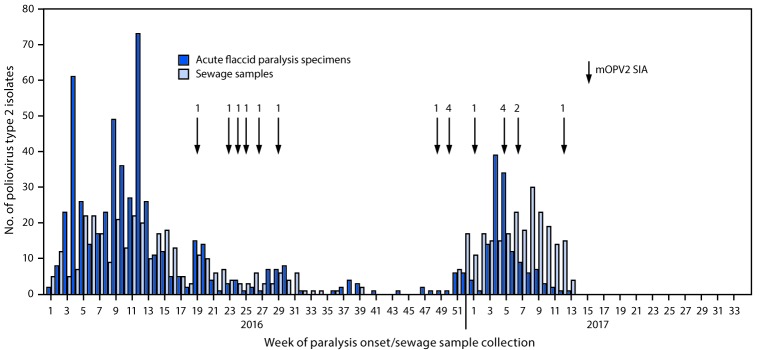
Number of poliovirus type 2 isolates from persons with acute flaccid paralysis or their contacts and from sewage samples in countries where mOPV2 SIAs were conducted* after the global synchronized switch from tOPV to bOPV — January 2016 to March 2017 **Abbreviations:** bOPV = bivalent oral poliovirus vaccine; mOPVs = monovalent oral poliovirus vaccine type 2; SIA = supplementary immunization activity; tOPV = trivalent oral poliovirus vaccine. * Number of vaccination rounds shown for SIAs.

During the pre-switch period (January–April 2016), PV2 was detected through both AFP and environmental surveillance; after the switch, PV2 was detected primarily through environmental surveillance (Figure 1) (Figure 2). In countries where mOPV2 was not used after the switch, few PV2 isolates were reported during September–December 2016, and 60% of the viruses detected were from sewage samples (Figure 1). Among 364 isolates detected in 2017, 228 (62.6%) were from sewage samples (Figure 1) (Figure 2).

To provide evidence concerning the origin and significance of circulating PV2, on August 1, 2016, GPLN laboratories began to refer all PV2s detected from all sources for genetic sequencing. Isolation of Sabin-like poliovirus with zero or few nucleotide differences from Sabin2 by GPLN laboratories were instrumental in 1) identifying continued use of tOPV in some countries post-switch and in 2) confirmation of three post-switch cVDPV2 outbreaks caused by genetically related cVDPVs that began circulating before the switch.

## Discussion

Virologic monitoring through AFP cases and sewage samples indicate that withdrawal of a live vaccine, OPV2, used in routine immunization programs and mass immunization campaigns, was successfully accomplished by the GPEI. Some evidence of limited use of tOPV after the global tOPV to bOPV switch was found; however, 1 year after OPV2 withdrawal, PV2 has been isolated only in the few areas where mOPV2 has been used in response to detection of cVDPV2 isolates. By expanding the preexisting surveillance network to include environmental surveillance for polioviruses during the last 5 years, GPLN successfully detected VDPV2 emergences and outbreaks to allow GPEI to respond in a timely manner. AFP and environmental surveillance with laboratory testing for poliovirus by GPLN will continue to play a long-term, critical role in ensuring polio eradication (*9*).

During the first year after the switch, although several emergences of VDPV2 occurred, including some in areas with low poliovirus immunity, such as Mozambique, only two new small-scale VDPV2 outbreaks were detected, in Sokoto, Nigeria, and Quetta, Pakistan, and mOPV2 was used to stop these outbreaks. However, it is noteworthy that ongoing persistent cVDPV2 transmission pre-switch was evidenced in Nigeria in April 2016 using environmental surveillance, and mOPV2 was used in Nigeria and in countries bordering Lake Chad (Cameroon, Chad, and Niger) to respond to this outbreak. Nigeria and Pakistan also have circulation of WPV1, and WPV1 circulation continues in Afghanistan.

Reintroduction of live PV2-containing vaccine through the use of 19 mOPV2 immunization campaigns to interrupt VDPV2 transmission in six countries (Cameroon, Chad, Mozambique, Niger, Nigeria, and Pakistan), from May 2016 to Mar 2017 has disrupted the goal of interrupting PV2 transmission globally after the switch. The GPEI has established a mOPV2 advisory group, which advises WHO about each use of mOPV2, after an in-depth review of risk assessments conducted after any VDPV2 event or outbreak detection. In countries where no type 2-containing vaccine has been used after the switch, only three countries (Russia, Iraq, and India) have reported VDPV2 detection since September 2016.

Environmental surveillance for polioviruses detected the majority of PV2 from September 2016 to March 2017. Detection and sequencing of polioviruses isolated from sewage samples is difficult because these isolates often represent complex mixtures of viruses. Despite these challenges, further expansion of environmental surveillance is needed to maintain the high level of vigilance required to detect and respond to any type 2 poliovirus from all sources in the future, including breaches in containment in facilities retaining or still working with PV2 materials, including WPV2.

PV2s were tracked in both human specimens and sewage samples using a newly designed molecular diagnostic assay and algorithm developed by CDC (real-time reverse-transcription–polymerase chain reaction assay for intratypic differentiation of polioviruses), which was rapidly and efficiently implemented in GPLN laboratories in 2016. PV2 detection and genetic sequencing has been essential for the following: 1) providing evidence of continued use of tOPV after the withdrawal of this vaccine in April 2016; 2) identifying and following up unusual patterns of PV2 detection or circulation that signal gaps in herd immunity against PV2; and 3) classifying VDPV2s as either circulating viruses (cVDPV2s) or originating from immunodeficient persons (iVDPV2s), or of ambiguous origin (aVDPV2s) (*10*). The lessons learned and the innovative mechanisms used to monitor and respond to any detection of PV2 from all sources will be leveraged to monitor type 1 and 3 polioviruses after WPV1 eradication and bOPV cessation.

**Summary**

**What is already known about this topic?**

The Global Polio Eradication Initiative (GPEI) has made substantial progress since 1988; in 2016, only 37 wild poliovirus (WPV) type 1 (WPV1) cases were detected, the lowest number ever recorded. WPV type 2 has been eradicated, and WPV type 3 has not been detected since 2012. To reduce the risk for paralysis from infection with vaccine-derived polioviruses (VDPVs), in April 2016, all 155 oral poliovirus vaccine (OPV)–using countries switched from trivalent OPV (tOPV) to bivalent OPV (bOPV), containing vaccine virus types 1 and 3.

**What is added by this report?**

After the withdrawal and destruction of tOPV, the GPEI devised mechanisms to monitor disappearance of type 2 polioviruses (PV2s) in human populations and the environment. Enhanced environmental surveillance and provision of clear guidance to the Global Polio Laboratory Network has allowed timely, accurate, and comprehensive detection of PV2 by examining approximately 208,000 stool specimens and sewage samples. Preceding the tOPV to bOPV switch (January–April 2016), 43 countries reported detection of PV2; during January–March 2017, the number of countries reporting PV2 had declined to seven.

**What are the implications for public health practice?**

To prevent paralysis caused by VDPVs, elimination of vaccine viruses from the environment will be critical. Lessons learned from surveillance for PV2 after the global synchronized withdrawal of the PV2 component from vaccines have resulted in development of standardized procedures for investigation of PV2 detection in humans and the environment, and handling PV2 in diagnostic laboratories. These lessons will guide the elimination of OPV1 and OPV3 once eradication of polio has been certified.
